# Nine Letters with Replies

**Published:** 1874-10

**Authors:** 


### Letters Exposing Humbugs and Swindlers,

**With other Communications of Interest to the People.**

ROCHESTER, N. Y., July 20, 1874.

DR. UP DE GRAFF.

*Dear Doctor:*—The BISTOURY is at hand, as lively and brimfull of useful information, as ever. I take six medical journals, at large cost, and I would rather part with every one of them than the BISTOURY. Enclosed two more names for it, with the necessary subscription. By the way, why is it that we have so many quack doctors traveling about the country, in view of the recent enactment of our legislature? Is it possible they are all licensed? If so, where is the good of such a law? W.

Thanks for the kind words of BISTOURY. With reference to the new law "regulating the practice of medicine," see editorial upon the subject.

---

EAST SMITHFIELD, Pa., Aug. 17, '74.

DR. UP DE GRAFF:

How old must a baby be before you can straighten its feet. We have a baby, real well and hearty, three months old, with one foot very crooked—turns in, so that were it to stand up, it would rest upon the outside of its foot. How long must we wait before doing something for it? Mrs. M.

A baby is never too young to have a club foot straightened. If you attend to it immediately, it can usually be accomplished without cutting. Proper strapping, under the eye of a skillful surgeon should be commenced at the earliest possible moment.

---

OVID, N. Y., Aug. 21st, 1874.

DR. UP DE GRAFF:

We have a child three years of age, a little boy, stout and hearty, but who *snores* terribly when asleep. Sometimes we are afraid he will choke. What can be the cause of it? We notice lately, he is growing a little deaf. What do you advise? J. P. R.

The snoring is caused, likely, by enlarged tonsils, which also could be the reason of his deafness. Better have him examined and prescribed for, else may the deafness become difficult of cure.

BLOSSBURG, Pa., July 6th, '74.

EDITOR BISTOURY:

We are told to write you for information relative to our mother. She is 52 years of age, has been very well, until recently she has had much pain in her eyes and eyebrows, accompanied by dimness of vision. Has flashes of bright light, and then great pain and tenderness of the eyeballs. The eyes are not inflamed, nor can we see anything wrong, except that they are a little larger than natural. Her vision has been very dim for two or three days and she is afraid she is going blind. Can you tell us what to do? M. A. R.

The symptoms are those that accompany what is known to the ophthalmic surgeon as *glaucoma*, a very serious form of blindness. If this is her real condition, nothing but an *iridectomy* (removing a portion of the iris), will save her from total and incurable blindness. Not a day should be lost in seeking this relief. We have so informed you by letter.

---

ST. JOSEPH, Mo., Sept 1st, '74.

THAD. S. UP DE GRAFF, M. D.:

A neighbor who reads your BISTOURY, advises us to write you for information relative to our son, aged 19, who is suffering from an abcess of his liver, at least that is what the doctors all say is the trouble. Our family physician advises that a surgeon be sent for and an instrument introduced into his liver and the matter taken out. Some say it can be done, and some say not. What we want to know is, whether such an operation is justifiable,—is it ever done? * * * * * * * Please answer immediately. P. W.

Yes, such a proceedure is justifiable, and if the *aspirator* is used, perfectly safe. It is not an uncommon occurrence that from six to eight pints of matter is obtained from the liver, through *aspiration*.

---

WILLIAMSPORT, Pa., Aug. 30th, '74.

DR. UP DE GRAFF:

I have a little boy, 5 years old, who complains with great pain, at times, in his hip. It does not seem to be sore to the touch, but he comes in from play, lies down and says his leg pains him. He is rather delicate, we think scrofulous, and the pain is growing upon him. Our physician says he thinks he will have

"hip joint disease." What should we do for him? B.

His symptons point strongly to what your physician suggests. Take him at once to a competent surgeon and have an "extension apparatus" applied. If the disease has not advanced too far, he will recover without resorting to an operation. See last BISTOURY for information upon this subject.

---

BOWLING GREEN, Ky., Aug. 18th, '74.

EDITOR BISTOURY:

I saw the BISTOURY yesterday, saw your answers to correspondents, and venture to ask for information of one John B. Ogden, Station D., Bible House, N. Y., who advertises to send a formula, free, that will cure debility, etc. I wrote him and he replied that he could send me the medicine cheaper than I could have it put up here, that he would send me a package for $3.65, and that three or four such packages would cure me. I thought I would drop you a line before I made the investment.

T. H.

We have exposed the swindling operations of John B. Ogden and those of his ilk, more than one hundred times during the past ten years, in the columns of the BISTOURY. We had supposed that no one remained, at this late day, green enough to invest a penny with any of the free-formula chaps. Their business has not prospered much for the past two years, and many of them have gone into bogus watch and counterfeit money operations. If you want to be easily swindled, send John your money—he'll do it up for you.

---

PHILADELPHIA, Pa., Sept. 18, '74.

Dr. UP DE GRAFF:

Enclosed subscription to BISTOURY. Have you seen Ziemssen's Cyclopedia of the Practice of Medicine? Am told there are 15 volumes at $5 a volume. Would like such a work if it *is* what they claim for it. Can you tell me anything about it? T. W.

The work is being translated under the supervision of Dr. Buck, of New York, and will be published by Wm. Wood & Co. of that city. The first volume is just issued. It promises to be an excellent work.

---

POUGHKEEPSIE, N. Y., Sept. 2, '74.

Dr. UP DE GRAFF:

Dr. —— has informed me of your general operating case of instruments, says they are compactly and neatly arranged. Will you be kind enough to give me the name of the manufacturer? J. R. C.

Geo. Tieman & Co., 67 Chatham St., N. Y., make our surgical instruments.

